# A School-Based Weekly Iron and Folic Acid Supplementation Program Effectively Reduces Anemia in a Prospective Cohort of Ghanaian Adolescent Girls

**DOI:** 10.1093/jn/nxab024

**Published:** 2021-06-01

**Authors:** Lucas Gosdin, Andrea J Sharma, Katie Tripp, Esi Foriwa Amoaful, Abraham B Mahama, Lilian Selenje, Maria Elena Jefferds, Reynaldo Martorell, Usha Ramakrishnan, O Yaw Addo

**Affiliations:** 1 Nutrition and Health Sciences, Laney Graduate School, Emory University, Atlanta, Georgia, USA; 2 Nutrition Branch, Division of Nutrition, Physical Activity, and Obesity, Centers for Disease Control and Prevention, Atlanta, Georgia, USA; 3 US Public Health Service Commissioned Corps, Atlanta, Georgia, USA; 4 Ghana Health Service of Ministry of Health, Accra, Ghana; 5 UNICEF-Ghana, Accra, Ghana; 6 Hubert Department of Global Health, Rollins School of Public Health, Emory University, Atlanta, Georgia, USA; 7 Emory Global Health Institute, Atlanta, Georgia, USA

**Keywords:** adolescent anemia, hemoglobin, adolescent females, iron, folic acid, supplementation, weekly iron supplementation, supplementation in schools

## Abstract

**Background:**

School-based iron and folic acid (IFA) supplementation is recommended for adolescent girls in countries with high burdens of anemia.

**Objectives:**

We aimed to evaluate the context-specific effectiveness of a school-based, integrated anemia control program with IFA supplementation in Ghana.

**Methods:**

Using data from a pre-post, longitudinal program evaluation, we evaluated the effectiveness of school-based weekly IFA supplementation in reducing the burden of anemia and increasing hemoglobin concentrations (Hb; primary outcomes) in 2 regions of Ghana. Generalized linear mixed effects models with schools (clusters) as random effects were used to quantify the change in the anemia prevalence and the mean Hb associated with cumulative IFA tablet consumption over 1 school year (30–36 weeks), controlling for participant-level potential confounders. A cut point for minimum effective cumulative IFA consumption that is reflective of adequate Hb was derived following logistic regression. This cut point was verified by a restricted cubic spline model of IFA consumption and Hb.

**Results:**

The analytical sample included 60 schools and 1387 girls ages 10–19 years. The prevalence of anemia declined during 1 school year of the intervention, from 25.1% to 19.6% (*P* = 0.001). Students consumed a mean of 16.4 IFA tablets (range, 0–36). IFA consumption was positively associated with Hb and negatively associated with anemia. Each additional IFA tablet consumed over the school year was associated with a 5% (95% CI, 1–10%) reduction in the adjusted odds of anemia at follow-up, though the relationship is nonlinear. The cut point for minimum effective consumption was 26.7 tablets over a 30–36-week school year, with tablets provided weekly.

**Conclusions:**

School-based weekly IFA supplementation is effective in improving Hb and reducing the anemia prevalence among schoolgirls in Ghana, though most participants consumed fewer than the minimum effective number of IFA tablets. Increasing intake adherence may further improve anemia outcomes in this population. *J Nutr* 2021;00:1–10.

## Introduction

Globally, iron-deficiency anemia is a leading cause of morbidity and mortality among adolescent girls 10–19 years of age (1). Anemia is defined by a low circulating hemoglobin concentration (Hb), a protein critical to the transport of oxygen (2). Anemia has negative consequences in the short term, such as reduced immune function (3), aerobic capacity (4), and metabolism (5), which can lead to increased illnesses, lethargy, fatigue, and lack of concentration (6). In the intermediate and long term, iron-deficiency anemia is associated with reduced work capacity (7) and cognition (8), which can lead to reduced human capital (6) and lost academic potential (9). Anemia during pregnancy can lead to poor outcomes, such as hemorrhage, preterm birth, low birth weight, perinatal mortality, and neonatal mortality (10).

Anemia has a complex and context-specific etiology (11). Blood disorders; micronutrient deficiencies; parasitic infections, including helminths and malaria; overweight/obesity; and blood loss (including menstruation) all contribute to the burden of anemia throughout the world (6, 12). In Ghana, adolescents carry a high burden of micronutrient deficiencies related to anemia. For example, in the national micronutrient survey conducted in 2017, 26% of girls 15–19 years old had anemia, 15% had iron-deficiency anemia, and 57% had folate deficiency (13). Our 2019 survey of in-school adolescents in 3 regions found that the prevalence of anemia among girls 10–19 years of age was 22%, significantly higher than the prevalence among boys of the same age (13%).

Schools have been identified as important delivery platforms for nutrition interventions among adolescents (14). A recent meta-analysis using data from 15 studies in 7 different countries showed that supplementation with iron and folic acid (IFA) tablets through schools may reduce the prevalence of anemia among adolescent girls (15). The WHO recommends intermittent IFA supplementation to all women during pregnancy; women of reproductive age (15–49 years of age), including adolescents; and school-aged children (5–12 years of age) where the prevalence of anemia exceeds 20%, and paired with malaria control measures where endemic (16, 17). However, few countries with high rates of anemia have a program in place for adolescents, and the African continent has very limited experience with this type of program (18). Schools may be an effective delivery platform in Ghana, since secondary school attendance has been increasing. In 2018, nearly half of girls 12–14 years (49%) were enrolled in junior high school and 30% of girls 15–17 years were enrolled in senior high schools, up from 25% in 2016 (19). With free public senior high school introduced in 2017, enrollment is expected to further increase.

We hypothesized that routine weekly supplementation with IFA in schools improved Hb and reduced anemia among adolescent schoolgirls in Ghana. This study aimed to measure the effectiveness of weekly school-based IFA supplementation on anemia status and Hb, and to determine a minimum effective number of IFA tablets associated with a reduction in anemia prevalence over a 30–36-week school year, with tablets provided weekly, in a prospective cohort of Ghanaian adolescent schoolgirls.

## Methods

### Program

The Girls’ Iron–Folic Acid Tablet Supplementation (GIFTS) Program is an integrated health and nutrition education program with IFA supplementation for the control of anemia among adolescent girls in Ghana. The program was rolled out in 3 phases. At the beginning of the school year in October 2017, Phase I of the program began in 4 regions: Brong-Ahafo, Northern, Upper East, and Volta. In December 2018, Ghana’s 10 regions were divided into 16 regions. Northern became Savannah, Northern, and North East. Volta became Volta and Oti. The mandate of the program was to reach all eligible girls aged 10–19 years, which disallowed the creation of a control group within Phase I regions. Use of nonimplementing regions as a comparison for the Phase I evaluation was judged as weak due to heterogeneity between regions in terms of population density infrastructure and climatological zones. The program has since been expanded to all regions of Ghana as a national program. The main delivery platform for the intervention is junior high, senior high, and technical/vocational schools, reaching approximately 400,000 girls. Though the program follows the WHO recommendations, benchmarks for program adherence have not been evaluated. There is existing evidence of a dose-response relationship between IFA supplementation and Hb; however, because of the downregulation of iron absorption in iron repletion (20), there may be a point at which this relationship plateaus, which could be interpreted as the minimum effective number of IFA tablets provided weekly over 1 school year needed for adequate Hb in this population. This could be used as a program metric for determining effective adherence.

### Study design and setting

A program effectiveness evaluation was designed as a pre-post, longitudinal study with a cohort of girls and schools from 2 of the Phase I regions, nested within the implementation of the GIFTS Program over 1 school year. All students received key messages about anemia risk factors, the consequences of anemia, prevention of anemia, prevention of malaria, water, sanitation, consumption of an iron-rich and diverse diet, and the use and benefits of IFA tablets. Adolescent girls received weekly supplementation with an IFA tablet containing 182.4 mg ferrous fumarate (60 mg elemental iron) and 0.40 mg folic acid through directly observed therapy. Of note, this formulation was used because tablets with 2.8 mg folic acid, as recommended by the WHO, were unavailable for purchase (17). IFA tablets were given on a single weekday by educators, but schools were instructed to provide makeup opportunities to girls absent on distribution day. Weekly IFA consumption data were recorded for each adolescent girl into classroom registers over the school year (30–36 weeks depending on the school).

For the nested evaluation, the Volta and Northern regions were purposively selected by the Ministry of Health from the 4 Phase I regions based on their high and low participation in other nutrition programs, respectively. Within each region, 15 junior high schools (JHS) and 15 senior high schools (SHS) were selected using probability proportional to size sampling from a list of all secondary schools (public and private) and their previous year’s enrollment of girls. The sampling frame included 837 JHS and 68 SHS in the Northern region and 1225 JHS and 104 SHS in the Volta region. In total, 60 schools were selected. To detect a 10% minimum reduction (equivalent to a 4–percentage point change) based on an estimated background prevalence rate of 40% anemia at a fixed power of 80% and a 2-sided 95% significance level, a sample of 1552 was required. A background prevalence of 40% was used because the most recent data available at the time of the survey planning was the Ghana Demographic and Health Survey 2014, which indicated a prevalence of 48% among adolescent girls aged 15–19 years (21). At the beginning of the 2017–2018 school year (September/October 2017), prior to the start of the program, 29 girls in each school were selected for the evaluation using simple random sampling. The program began immediately following baseline data collection. At the end of the school year (after 8 months, in June 2018), participating girls were followed up.

Written consent from parents and verbal consent from students were obtained. Procedures followed were in accordance with the ethical standards of the Ghana Health Service ethics committee, in accordance with the Helsinki Declaration of 1975 as revised in 1983.

Questionnaires were administered to selected adolescent girls and to a representative from each selected school by trained enumerators using tablet-based electronic data collection. School representatives were the focal person (primary implementer) for the GIFTS program, typically the school health education and promotion coordinator. Questionnaires consisted of demographic characteristics, diet, and knowledge, attitudes, and practices related to anemia and IFA. Dietary intake was assessed over the previous 24 hours using a modified FFQ emphasizing sources of dietary iron (22). Height and weight were collected following standard anthropometric methods (23). Capillary blood was used to measure Hb using a HemoCue 301 and malaria antigens using the CareStar Rapid Diagnostic Test (AccessBio). During the follow-up survey, the number of IFA tablets consumed by each selected girl was abstracted from classroom registers, and questionnaires collected additional information on program experiences from students and teachers.

### Study variables

Household wealth was calculated following a principal components analysis of household assets, and households were divided into tertiles. The school was classified as rural, peri-urban, or urban using national census data. Food frequency data were categorized based on iron content or relevant interactions (i.e., inhibitors or enhancers of iron absorption) (24). Rich sources of heme iron included red meats and organ meats. Fair sources of heme iron included poultry, pork, fish, and eggs. Rich sources of nonheme iron included dark green leafy vegetables and legumes. Foods and beverages fortified with iron included locally available cereals and beverages with an iron fortificant on the label. Although mandatory, wheat flour fortification is poor; only 13% of samples in a 2011 survey were fortified to the required standard (25). Accordingly, we did not group wheat flour products with fortified foods and beverages. Benchmarks set by stakeholders were used to describe coverage and intensity of the intervention: *1*) taking at least 1 IFA tablet; and *2*) taking at least 10 IFA tablets over the school year. The cumulative number of IFA tablets consumed was categorized within each term and over the school year.

BMI-for-age z-scores (BMIZ) were calculated following International Obesity Taskforce age-specific parameters, and cutoffs for thinness (BMIZ < −2 SD), overweight (+1 SD < BMIZ ≤ +2 SD), and obesity (BMIZ > +2 SD) were applied (26). Thinness (<1% of sample) was grouped with normal weight for further analyses. Malaria results were dichotomized as positive or negative. Participants with Hb < 10 g/dL or a positive malaria result were referred for treatment at their local health facility and remained in the evaluation. Anemia, the primary outcome variable, was defined using age-specific Hb cutoff values (10–11 years: Hb < 11.5 g/dL; ≥ 12 years: Hb < 12 g/dL) (2). No adjustment for altitude or smoking was performed since all populations lived at elevations below 1000 meters (2) and smoking is extremely rare among adolescent girls in Ghana (27).

### Statistical analysis

CIs and *P* values for comparisons of continuous data were calculated using complex survey procedures with Taylor series variance, where appropriate. Rao-Scott chi-square tests were used to test for differences in proportions. These procedures were used to account for the clustering of the data. Mean and prevalence differences were calculated between baseline and follow-up for health and dietary characteristics of study participants. To understand the factors that influenced the changes in Hb and anemia, generalized linear mixed effects models (GLMMs) were fit to anemia prevalence using a logistic link function and to Hb using a linear link function, accounting for clustering of girls at the school level as random effects with regions as strata. The GLMMs made use of all the data from baseline and follow-up using maximum likelihood estimation. Models included the cumulative number of IFA tablets consumed over 1 school year and potential confounders as fixed effects, including demographics (age and wealth tertile), school characteristics (level and rurality), health (malaria, BMI category, and geophagy), and diet (sources of iron and citrus). Health and dietary variables were included as time-dependent predictors. Categories of cumulative IFA consumption were based on program targets of a minimum of 10 weeks of supplementation for the school year; therefore, groups of 10 IFA tablets were used to capture gradations of consumption. The 1–10 group was the reference group for multivariable analysis because of a more robust sample size in comparison to the 0-dose group. Adjusted prevalence and mean Hb values were computed from the model for each time period. Significance was determined from the Type III test of the IFA tablet categories as fixed effects with 4 degrees of freedom. Collinearity was assessed by variance inflation factor, after which menarche was excluded as a covariate for collinearity with age. We examined an effect modification between cumulative IFA tablets consumed and the change in anemia prevalence difference by other model covariates.

Having examined the association between IFA tablets consumed and Hb or anemia, an exploratory data analysis was conducted to examine the dose-response relationship between them and identify a minimum effective number of IFA tablets provided weekly over 1 school year. In order to determine the minimum effective number of cumulative IFA tablets provided weekly over 1 school year, binary logistic regression was used, modeling the probability of no anemia at follow-up and the cumulative number of IFA tablets consumed over 1 school year; controlling for potential confounding variables, including demographics (age and wealth tertile), school characteristics (level and rurality), health (malaria, BMI category, and geophagy), and diet (sources of iron and citrus); and accounting for clustering. We used receiver operating characteristic curve analyses and Youden’s Index to maximize the sensitivity and specificity of the cut point chosen (28). The fitted logistic function was solved for the minimum effective number of cumulative IFA tablets for identifying the predicted probability of no anemia at the point of maximum diagnostic effectiveness, identified by Youden’s Index. Precision around the minimum effective cut point was derived from a percentile bootstrap (1000 replicates) estimation. Sampling weights were applied where appropriate. A priori, alpha was set at 0.05. All analyses were conducted in SAS 9.4 (SAS Institute) unless otherwise noted. Additionally, a restricted cubic spline (RCS) model was built to examine linear and curvilinear trends in the relationship between cumulative IFA consumption and the change in Hb over the school year. The number of knots was automatically chosen based on model fit and empirical distribution. This analysis was conducted in R version 3.5.1 (R Foundation for Statistical Computing).

## Results

**FIGURE 1:**
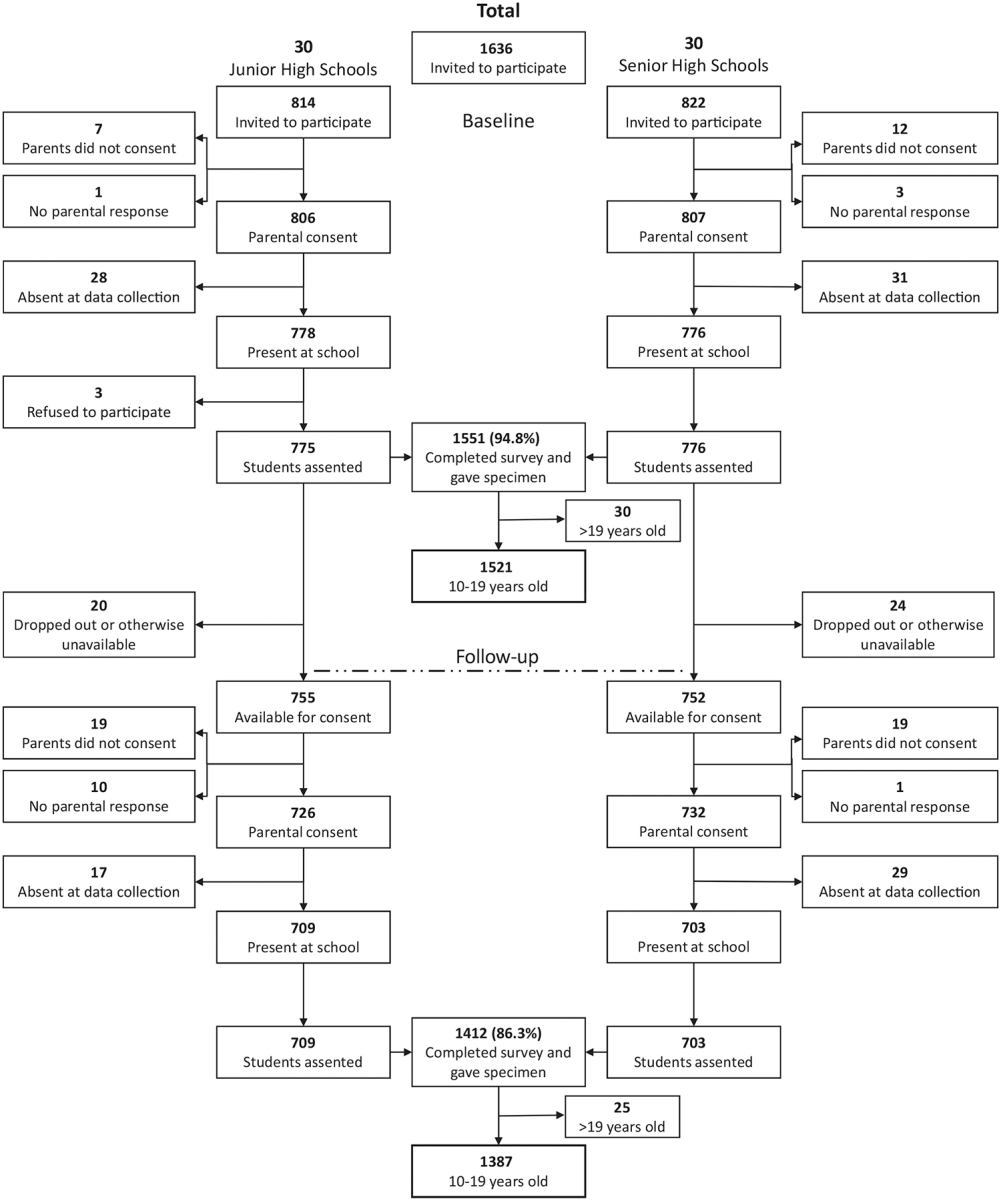
Participant flow chart for baseline and follow-up surveys of adolescent schoolgirls in the Northern and Volta regions of Ghana. Follow-up occurred 9 months after baseline.

A total of 1551 selected girls (94.8%) agreed to participate and completed the baseline survey; at follow-up, 1412 participated again (91.0%; Figure 1). We excluded 30 students who were older than 19 years at baseline, for a final analytical sample of 1521 at baseline and 1387 at follow-up. At baseline, the median age of participants was 15.3 years (range, 10–19 years), and most had reached menarche (84.7%). Students primarily attended government schools (93.3%) located in rural settings (53.5%; Table 1). The 134 students lost to follow-up were a mean of 6 months older (*P* = 0.03) but did not differ from those who remained in terms of other demographic or health characteristics (data not shown).

There were significant differences between the baseline and follow-up in the health and dietary characteristics of participants. The proportions of girls with anemia and malaria significantly declined over time (−5.4 percentage points and −17.7 percentage points, respectively), with a corresponding increase in mean Hb (+0.2 g/dL). Over the 2 time points, there were also significant increases in the proportion that reported consuming sources of iron and citrus fruits. There were no significant changes in body composition or the proportion engaging in geophagy (Table 2). Among girls who had anemia at baseline, 51.0% (95% CI, 43.3–58.6) no longer had anemia at follow-up, and among those without anemia at baseline, 9.5% (95% CI, 6.0–13.1) developed anemia by the follow-up (*P <* 0.0001; table not shown).

**FIGURE 2:**
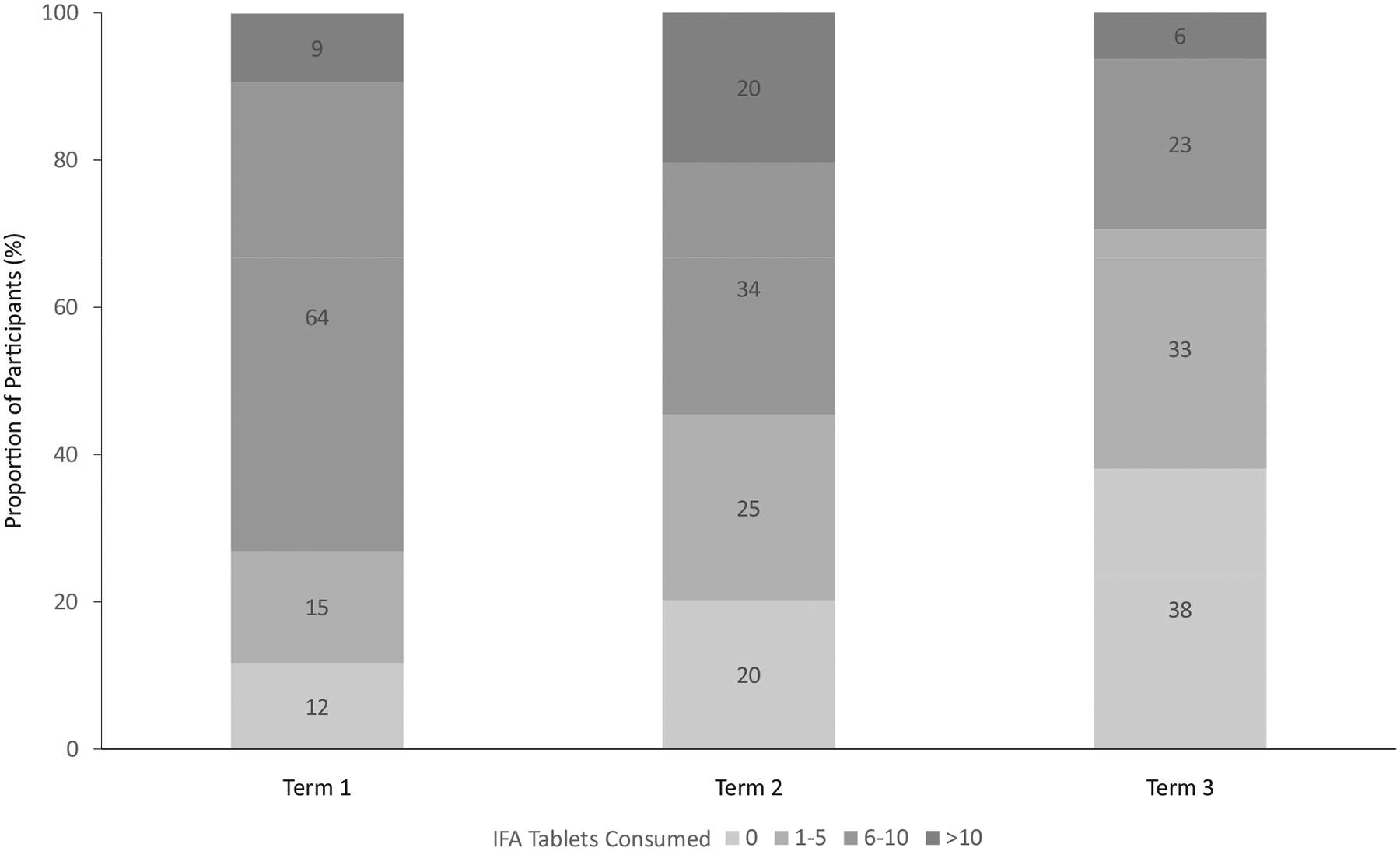
IFA tablets consumed among adolescent schoolgirls in the Northern and Volta regions of Ghana over 1 school year by term (*n* = 1387). Proportions are weighted to be representative of all eligible girls in the school. The school year is divided into 3 terms, the first of which began with the baseline and the third of which ended 9 months later with the follow-up survey. Grand total of IFA tablets consumed: 28,005. Mean (minimum, maximum) number of IFA tablets consumed: 16.4 (0, 36). Abbreviation: IFA, iron and folic acid.

A total of 28,005 IFA tablets were consumed by the study participants over the study period during the 2017/18 academic year (October 2017–June 2018), a mean of 16.4 tablets per girl (range, 0–36; Figure 2). In terms of program coverage and adherence, most students (89.5%) had consumed at least 1 tablet during the school year, and nearly 75% had taken more than 10 tablets. The school year is made up of 3 terms lasting approximately 12–15 weeks each, and follow-up data were collected approximately 2 weeks before the end of the final term. The cumulative number of IFA tablets consumed varied by term, with more students consuming 6 or more tablets during term 1 than in the remaining 2 terms (*P <* 0.0001; Figure 2).

**FIGURE 3:**
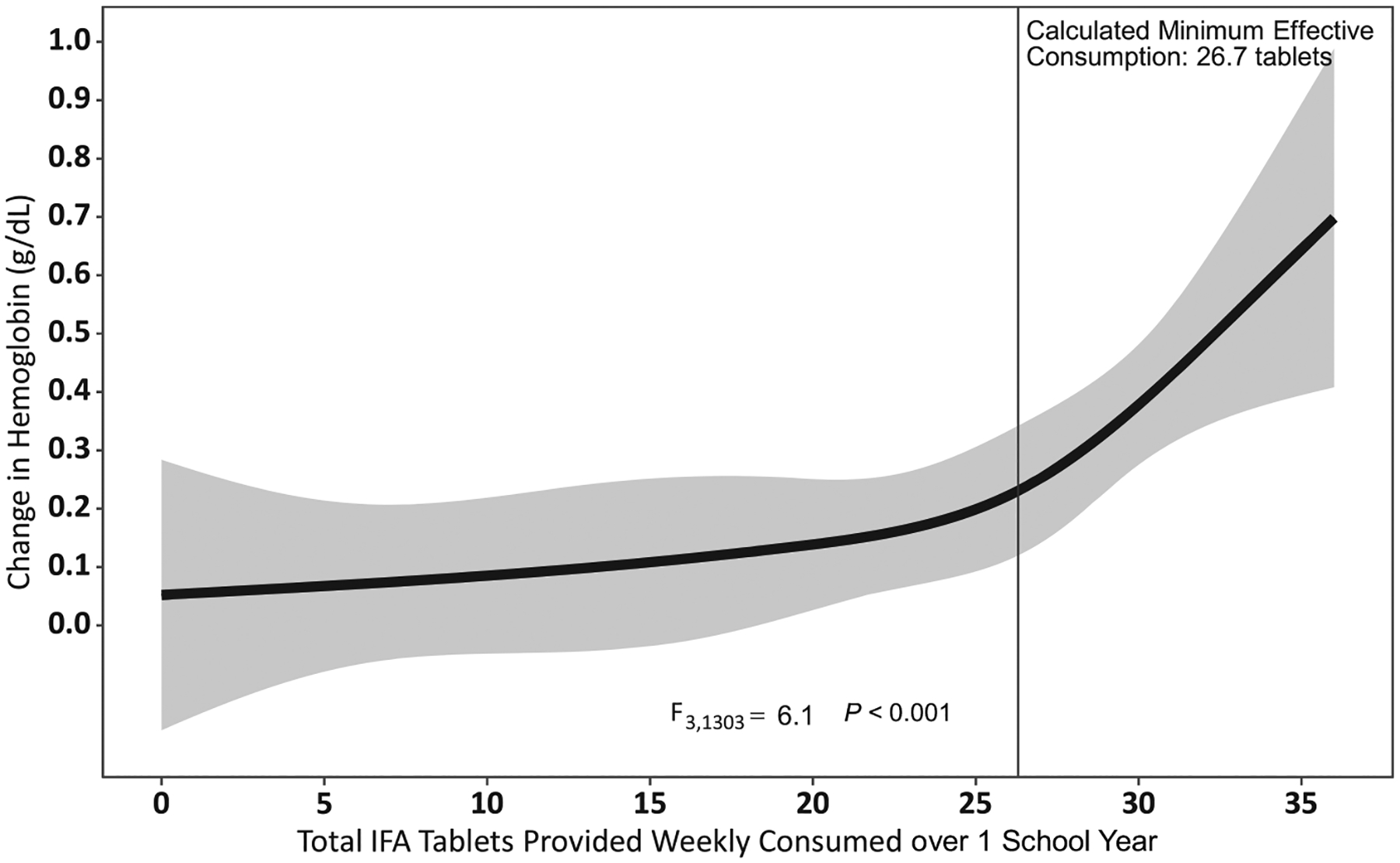
The relationship between the cumulative number of IFA tablets consumed and the change in hemoglobin concentration over 1 school year among adolescent schoolgirls in the Northern and Volta regions of Ghana (*n* = 1307). Restricted cubic spline model with knots at 0, 20, 27, and 33 tablets. Abbreviation: IFA, iron and folic acid.

The RCS model had 4 knots at the 5th (0 tablets), 35th (20 tablets), 65th (27 tablets), and 95th (33 tablets) percentiles and shows that there is a positive, nonlinear relationship between IFA dosage and change in Hb (*P <* 0.0001; Figure 3). For IFA consumption between 0–20 tablets, each additional tablet consumed was associated with a near 0 increase in Hb (0.003 g/dL); whereas for consumption between 27–33 tablets, each additional tablet was associated with a 0.17 g/dL increase in Hb.

**FIGURE 4:**
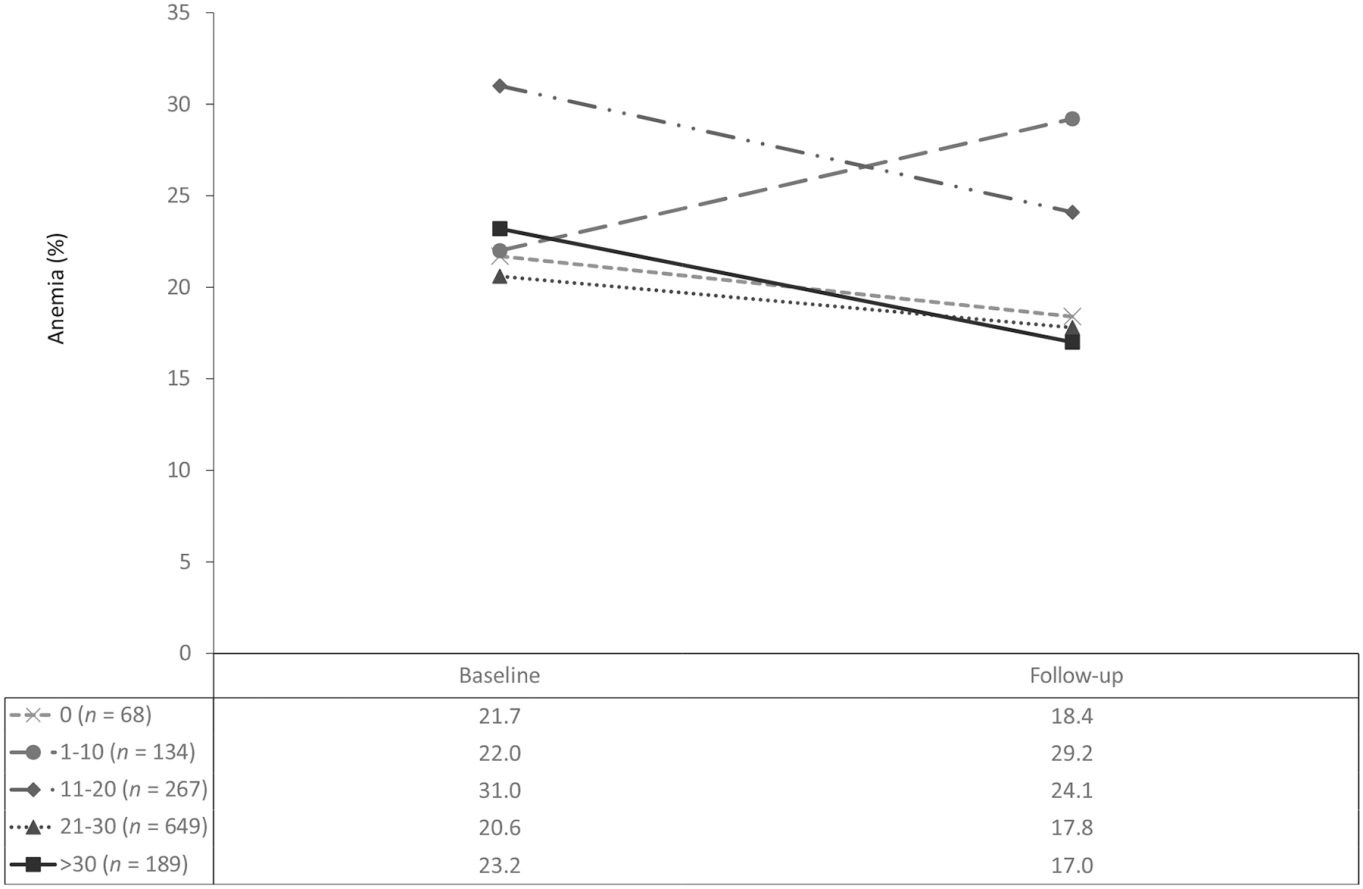
Adjusted changes in anemia prevalence differences by cumulative IFA tablets consumed over 1 school year among adolescent schoolgirls in the Northern and Volta regions of Ghana (*n* = 1307). Follow-up occurred 9 months after baseline. Estimates are weighted to be representative of all eligible girls in the school. Estimates and *P* values are calculated from generalized mixed models using maximum likelihood estimation, and accounted for clustering in school and intra-individual covariance. Adjusted for demographics, school level and geography, health characteristics, and diet. *P* value for trend = 0.024. Abbreviation: IFA, iron and folic acid.

**FIGURE 5:**
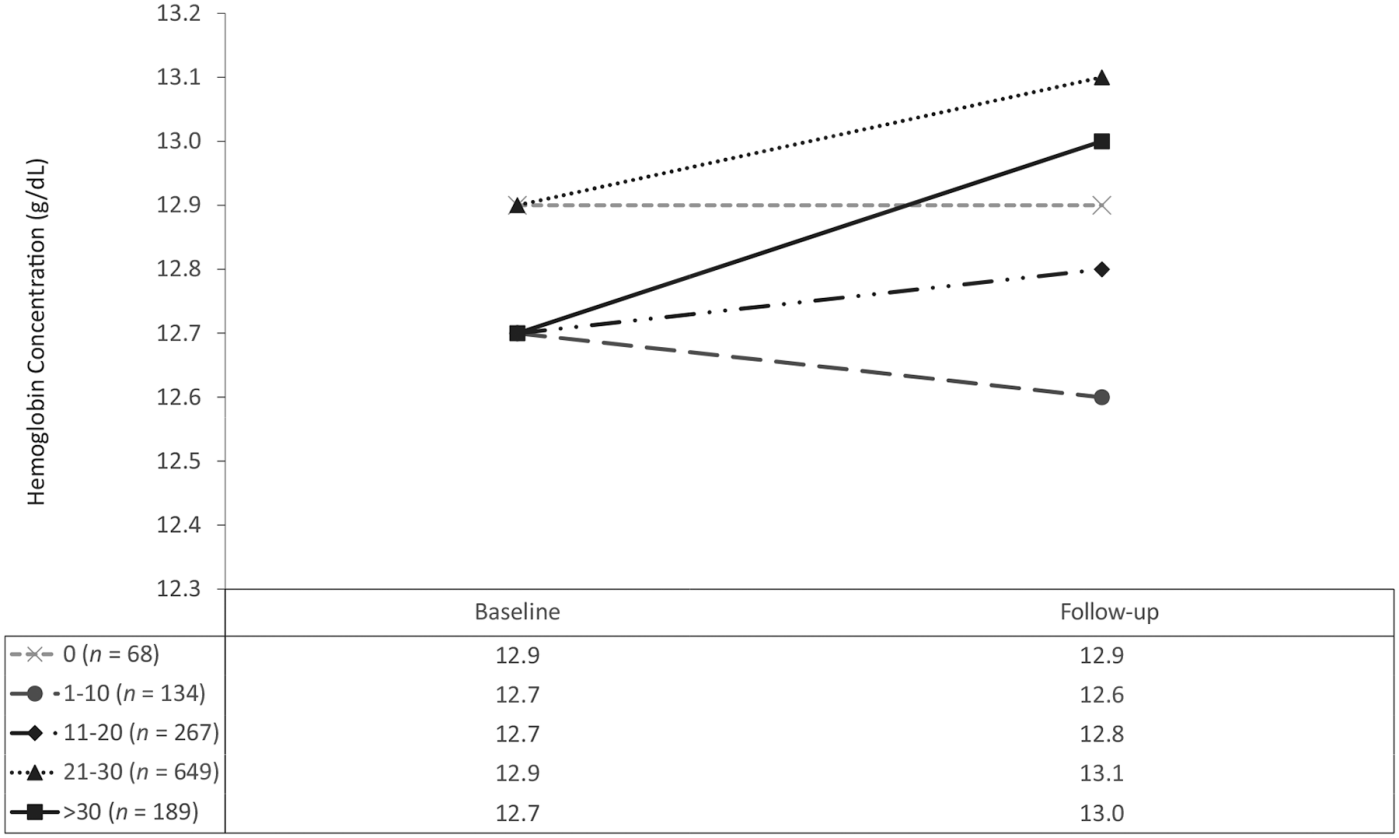
Adjusted changes in mean hemoglobin differences by cumulative IFA tablets consumed over 1 school year among adolescent schoolgirls in the Northern and Volta regions of Ghana (*n* = 1307). Follow-up occurred 9 months after baseline. Estimates are weighted to be representative of all eligible girls in the school. Estimates and *P* values are calculated from generalized mixed models using maximum likelihood estimation, and accounted for clustering in school and intra-individual covariance. Adjusted for demographics, school level and geography, health characteristics, and diet. *P* value for trend = 0.029. Abbreviation: IFA, iron and folic acid.

In the GLMM, the group of girls who had never taken an IFA tablet had an adjusted decrease in the anemia prevalence (21.7 to 18.4%). This group had among the highest baseline Hb values and were more likely to have been in SHS, have reached menarche, and have consumed iron-fortified foods (data not shown). In fact, none of the girls who had never consumed IFA tablets reached menarche during the intervention, compared to 71 (2.1%) of the girls who had consumed IFA tablets. However, these groups did not differ in age, household wealth, preintervention iron supplementation, other elements of the diet, or BMI category (data not shown). The adjusted prevalence of anemia increased among the group who had consumed between 1 and 10 tablets (from 22.0 to 29.2%), and their Hb decreased (from 12.7 to 12.6 g/dL). Changes observed in the 1–10-tablets group were not significantly different from those in the group who had never consumed a tablet (Supplemental Table 1). The adjusted anemia prevalence decreased and Hb increased for the remaining groups. The adjusted population prevalence of anemia (as least square marginals from GLMMs) decreased substantially in the group who had consumed 11–20 tablets (from 31.0 to 24.1%), and their Hb increased (from 12.7 to 12.8 g/dL). There were also declines in the prevalence of anemia for the groups that had consumed 21–30 and *>*30 tablets (from 20.6 to 17.8% and from 23.2 to 17.0%, respectively). Their Hb also increased (from 12.9 to 13.1 g/dL and from 12.7 to 13.0 g/dL, respectively; Figures 4 and 5). Except for the consumers of only 1–10 tablets, with each category of an increasing number of IFA tablets consumed, there was a corresponding increase in Hb (*P* = 0.029) and decrease in anemia (*P* = 0.024). There was evidence of possible effect modification by wealth and rurality in the relationship between the number of tablets consumed and the change in anemia prevalence; however, the estimates were small, unstable, and not practically meaningful (results not shown).

The GLMM suggests a dose-response relationship between the cumulative number of IFA tablets taken over 1 school year and Hb at follow-up. We formally tested for linear and nonlinear trends in the association between Hb change and IFA using RCS models, and found statistically significant nonlinear trends (*P <* 0.001; Figure 3). This nonlinearity demonstrated by the RCS model indicates there may be plateauing of the potential impact on Hb changes at certain IFA doses that may be useful for setting program benchmarks. Investigating this relationship further, a model of anemia at follow-up and the cumulative number of IFA tablets consumed (adjusted for baseline Hb, a squared term of cumulative IFA tablets to account for nonlinearity, demographics, health characteristics, and diet) has good predictive power, with 82.1% AUC (Table 3). The derived cut point for the population minimum effective number of cumulative IFA tablets consumed over 1 school year is 26.7 tablets (95% CI, 26.0–28.3). Results were similar using an unadjusted model (26.9 tablets). The change in Hb was 0.06 g/dL for girls who consumed no tablets relative to 0.27 g/dL for girls who consumed 27 tablets or more over the 8-month school year.

## Discussion

This longitudinal, pre-post study provides evidence for the effectiveness of school-based IFA supplementation in reducing the burden of anemia in a programmatic setting in Ghana. The prevalence of anemia decreased by 5.4 percentage points, and the mean Hb increased by 0.15 g/dL. Similar declines in anemia prevalence were observed in India’s adolescent IFA supplementation program (30% in pilot regions and 5–24% during national scale-up) (29, 30). A meta-analysis of randomized controlled trials of intermittent IFA supplementation among adolescents and women of reproductive age found a mean 35% reduction in the risk of anemia and a 0.52 g/dL increase in Hb (31). Although the designs are not directly comparable, the differences between these meta-analysis results and our program effectiveness evaluation are attributed to the real-world circumstances of a program being implemented by the Ministries of Health and Education rather than the controlled experimental conditions of the studies included in the meta-analyses (31). Specifically, many of the interventions that have reported evaluations have had a more limited reach and been implemented under controlled conditions or by researchers and Ministries of Health, whereas this evaluation reports on an intervention integrated into existing structures by both education and health sectors to the entire population of schoolgirls in the regions. The positive impact of this program on anemia is rather encouraging, particularly for countries seeking to embark on such interventions using existing platforms, because it shows improvements without the infeasible conditions of randomized controlled trials.

A reduction of 5.4 percentage points in the population prevalence of anemia is a decrease of approximately 22,000 cases of anemia among adolescent girls in schools within the surveyed regions over only 8 months. This effect, when extended to all 3 million adolescent girls in Ghanaian schools, might avert 165,000 cases of anemia, which is not trivial in a malaria-endemic region. Further, a reduction of 5.5 percentage points is also much larger than the approximate 0.025 percentage point annual decrease in anemia prevalence among nonpregnant women 15–49 years in the Central and West Africa region between 1995 and 2011 (32). While regional trends may differ from those of individual countries, this comparison highlights the dramatic decrease associated with this integrated IFA intervention. IFA supplementation may benefit consumers beyond measured improvements in anemia because iron and folate deficiencies, identified as prevalent issues, can exist before anemia is detected (13). Additionally, we observed a significantly higher prevalence of girls who were “cured” relative those who developed anemia during the 8-month program, as well as a significant increase in mean Hb, further supporting the internal consistency of findings.

Except for the small group of girls who had never consumed an IFA tablet, consumers of more tablets had a greater adjusted decrease in the prevalence of anemia and a greater adjusted increase in Hb over the study period. We observed a statistically significant departure from a linear dose-response relationship between IFA consumption and Hb, with plateau ranges beyond which there was sharp increase in the change in hemoglobin levels at about 27 IFA tablets (Figure 3). Similarly, there was an overall significant trend between categories of IFA consumption separately with anemia and hemoglobin from GLMMs (type-3 test). Although the overall trend persisted, pairwise comparisons of associations across IFA consumption categories (0, 1–10, 11–20, 20–30, >30) yielded inconsistent results, potentially due to unbalanced and/or smaller sample sizes. For example, the group of never-consumers had the smallest sample size (*n* = 68), resulting in unstable estimates of changes in anemia and Hb and wider CIs in comparison to the other categories. Further, because the biologic IFA-Hb relationship is curvilinear, it might not translate into linear changes in anemia across IFA consumption categories.

We wished to investigate the independent effects of consumption of IFA during the most recent school term (Term 3) on Hb/anemia; however, the imprecision of the estimates from this sensitivity analysis inhibited our ability to interpret findings. Further, our principal study design did not examine Hb by school term, which also limited our ability to investigate the rate of change during the school year.

To ensure appropriate care, 174 participants with moderate to severe anemia and 355 additional participants with malaria were referred to their local health center at baseline (35% of participants). In a sensitivity analysis, these participants were excluded to determine whether the outcomes resulted from health center referrals. Among those who were not referred, the prevalence of anemia declined significantly, from 20.9% to 16.5% (*P* = 0.035), and mean Hb remained unchanged. However, based on student reports at follow-up, only 37 students sought treatment since they were told they had anemia. Excluding these individuals does not change the overall estimates or the changes in anemia prevalence and mean Hb.

We found 26.7 weekly tablets over the school year may be the minimum effective number of IFA tablets over 1 school year in this population. This cut point is consistent with the nonlinear dose-response relationship identified by the RCS model. The WHO recommendation for intermittent IFA supplementation (3 months on, 3 months off) amounts to approximately 26 tablets per year, nearly identical to the cut point derived in our analysis (17). This cut point is more than double the current program benchmark of a minimum of 10 tablets over the school year. Because of a lack of experience with this type of program, this benchmark was set heuristically because it is approximately one-third of the weeks of the school year. Program benchmarks may need to be raised, given that there are additive increases in Hb levels with consumption greater than 26 tablets. While the large proportion of girls (76%) who had consumed at least 10 tablets over the school year shows that adherence to the current program benchmark is feasible, only 21% had consumed at least 27 tablets. This suggests that more than three-quarters of students are missing the full benefit of the program. Intake adherence is lower in this program than in similar IFA programs (33, 34), implying program bottlenecks. Bottlenecks such as supply chains, training, stakeholder buy-in, side effects, and perceptions have been identified by similar programs in other countries and may be responsible for lower adherence (29, 35, 36). In this evaluation, only 3% of schools missed a distribution of IFA tablets due to no supply, and other school-level factors, including teacher training, school level, make-up distributions, and teacher perceptions, were associated with adherence (37). Participants reported few side effects, though changes in menstruation were commonly linked by participants to consumption of IFA tablets. Nearly 88% of participants reported that they liked the IFA tablets (37). The results indicate that program effectiveness may be improved by targeting programmatic issues at schools and with teachers.

This study is representative of 2 regions of Ghana during the first year of the intervention. It is limited by the absence of a control group. Individual-level and district-level randomization were not feasible for such a program carried out through existing health and education frameworks. Instead, a pre-post design was used, nested within the implementation of a large program. Causal inference is limited in such a design because changes observed may not be due to the intervention but rather to some other factors that also changed over time. In this study, 3 key factors—diet, malaria status, and age—changed between baseline and follow-up. Diet and malaria status are key factors that may have been affected by the differing seasons between baseline and follow-up. Follow-up occurred during a somewhat lower malaria transmission season, which could have led to fewer cases of malaria-induced anemia (38). Baseline was conducted during the harvest season (39); however, there was an increase in consumption of iron-rich foods at follow-up, suggesting other contributors to these changes beyond food availability, such as the nutrition education component of the GIFTS program. We have presented adjusted models that account for changes in diet and malaria status. Adding to evidence of the intervention’s effect is the dose-response relationship observed between cumulative IFA consumption and changes in Hb. Still, the models are not exhaustive, as they lack other potential contextual and individual predictors of Hb, such as hemoglobinopathies, micronutrient deficiencies, and other illnesses, including parasitosis, which may be particularly relevant given the high prevalence of geophagy. However, since girls were followed longitudinally, the proportion with hemoglobinopathies remained unchanged, and the prevalence of geophagy also remained constant, meaning that this route of potential exposure to parasites did not diminish even as the prevalence of anemia did. We were also limited in the evaluation of program impact by the absence of biomarkers of iron and folate status, which may have illuminated the mechanisms by which the hemoglobin status was improved or additional benefits of the IFA tablets. The minimum effective cut point for cumulative IFA tablets provided weekly over 1 school year provides a data-driven target for the maximum population benefit, and the bootstrapped approach corrects for potential overfitting and optimism bias. However, the cut point is based on an adjusted model that, while having good predictive qualities, cannot perfectly predict anemia. Another limitation is that this method, based on hemoglobin, does not identify the number of tablets needed for achieving an adequate iron or folate status.

There is growing interest in supplementation of adolescents through schools. To our knowledge, Ghana is the first sub-Saharan country to implement IFA supplementation among adolescent girls and has the only active national program in Africa. While iron absorption is mediated by its bioavailability, gut integrity, iron stores, and infection, these results present a promising avenue for anemia control in this population.

## Conclusion

Supplementation with weekly IFA tablets in schools may have improved Hb and reduced anemia among adolescent girls in Ghana, independent of other measured contributors to the burden of anemia in this population. These effects, observed after a relatively short time, make IFA supplementation in schools a promising intervention for addressing the burden of anemia in Ghana. The calculated minimum effective number of IFA tablets suggests that improved intake adherence would improve the population benefit from the program. Program targets should be higher than the current target of 10 tablets over 1 school year. Further research is needed to understand the drivers of adherence to IFA supplementation for improving the program and its impact on anemia control and prevention.

## Supplementary Material

Supplemental Table 1

## Figures and Tables

**TABLE 1:** Demographic characteristics of participants in the baseline survey of adolescent schoolgirls in the Northern and Volta regions of Ghana

Characteristic	Baseline	
	*n*	Estimate (95% CI)^1^
Demographics		
Age, years	1521	15.7 (15.3–16.1)
Reached menarche, %	1289	84.7 (80.1–89.4)
School		
Level		
Junior high school, %	773	50.8 (37.6–64.1)
Senior high school, %	700	46.0 (32.8–59.2)
Vocational school, %	48	3.2 (0.0–7.7)
Type		
Private, %	102	6.7 (0.6–12.8)
Government, %	1419	93.3 (87.2–99.4)
Location		
Rural, %	813	53.5 (40.2–66.7)
Peri-urban, %	438	28.1 (16.1–40.1)
Urban, %	287	18.4 (8.2–28.6)

Data are unweighted. The 95% CIs are based on Taylor series variance estimates to account for the complex sampling design.

1 Values are means or % (95% CIs).

**TABLE 2:** Health and dietary characteristics at baseline and follow-up and the population mean changes over 1 school year among adolescent schoolgirls in the Northern and Volta regions of Ghana

Characteristic	Baseline		Follow-up		Difference^1^	
	*n*	Estimate (95% CI)^2^	*n*	Estimate (95% CI)^2^	Estimate (95% CI)	*P* value
Health						
Anemia, %	363	25.1 (21.8–28.4)	255	19.6 (16.5–22.8)	− 5.4 (−8.8 to −2.1)	0.001
Hemoglobin concentration, g/dL	1521	12.7 (12.6–12.8)	1387	12.9 (12.8–13.0)	0.2 (0.1–0.3)	0.001
Positive malaria rapid diagnostic test, %	504	26.1 (22.9–29.3)	210	8.4 (6.6–10.3)	− 17.7 (−21.0 to −14.4)	*<*0.001
Thinness (BMIZ < −2 SD), %	23	1.0 (0.3–1.6)	20	0.8 (0.3–1.3)	− 0.2 (−0.7 to 0.3)	0.484
Overweight (+1 SD < BMIZ ≤ +2 SD), %	221	18.9 (15.8–22.0)	211	20.0 (16.7–23.3)	1.1 (−1.5 to 3.7)	0.405
Obesity (BMIZ > +2 SD), %	32	3.0 (1.7—4.3)	35	3.4 (1.9–48)	0.3 (−0.4 to 1.1)	0.377
Practice geophagy (eating soil or clay), %	450	26.8 (23.5–30.0)	301	23.3 (19.8–26.7)	− 3.5 (−7.6 to 0.6)	0.095
Diet (consumption in previous 24 hours)						
Rich source of heme iron,^3^ %	264	17.3 (14.4–20.1)	370	24.0 (20.7–27.2)	6.7 (2.6–10.8)	0.001
Fair source of heme iron,^4^ %	1137	71.3 (67.7–74.9)	1142	78.6 (75.1–82.0)	7.2 (2.7–11.8)	0.002
Rich source of nonheme iron,^5^ %	1033	66.8 (63.2–70.4)	1115	71.9 (68.2–75.7)	5.2 (0.2–10.1)	0.042
Foods and beverages fortified with iron,^6^ %	449	41.6 (37.8–45.3)	605	54.1 (50.2–58.1)	12.6 (8.0–17.1)	*<*0.001
Citrus fruits,^7^ %	483	23.7 (20.6–26.8)	579	34.5 (30.8–38.2)	10.8 (6.1–15.6)	*<*0.001

Proportions and means are weighted to be representative of all eligible girls in the school. Follow-up occurred 9 months after baseline. Abbreviation: BMIZ, BMI-for-age z-score.

1 Differences are mean differences or prevalence differences. The 95% CIs and *P* values are based on Rao-Scott chi-square tests and ANOVA with Taylor series variance estimates to account for the complex sampling design. BMIZ was determined by the International Obesity Task Force reference population.

2 Values are means or % (95% CIs).

3 Red meats, such as beef, lamb, goat, or wild game and organ meats.

4 Other animal-source foods, including eggs, poultry, and fish.

5 Dark green leafy vegetables, legumes, nuts, and seeds.

6 Cereal and beverages fortified with iron Milo, Ovaltine, Cerelac, Yumvita, or Nido.

7 Citrus fruits, including oranges, lemons, pineapple, mango, etc.

**TABLE 3:** Adjusted model of anemia at follow-up among adolescent schoolgirls in the Northern and Volta regions of Ghana (*n* = 1307)

Variable	Adjusted OR (95% CI)
Cumulative number of IFA tablets consumed	0.95 (0.90–0.99)*
Cumulative number of IFA tablets consumed, squared^1^	1.00 (1.00–1.00)*
Baseline hemoglobin concentration, g/dL	0.33 (0.25–0.44)***
Age, years	1.17 (1.02–1.35)*
Wealth tertile	
Middle vs. lowest	0.60 (0.34–1.05)
Highest vs. lowest	0.34 (0.17–0.67)**
Overweight vs. normal weight	1.21 (0.63–2.31)
Obesity vs. normal weight	0.78 (0.19–3.23)
Practicing vs. not practicing geophagy	1.32 (0.77–2.27)
Positive vs. negative malaria test	1.40 (0.59–3.31)
Rich source of heme iron vs. not consumed^2^	1.23 (0.72–2.09)
Fair source of heme iron vs. not consumed^3^	1.07 (0.55–2.09)
Rich source of nonheme iron vs. not consumed^4^	1.54 (0.87–2.73)
Foods and beverages fortified with iron vs. not consumed^5^	1.50 (0.93–2.40)
Citrus fruits vs. not consumed^6^	0.89 (0.50–1.59)

Estimates are weighted to be representative of all eligible girls in the school. Estimates, 95% CIs, and *P* values are calculated using multivariable logistic regression with Taylor series variance and account for clustering in school and intra-individual covariance. Follow-up occurred 9 months after baseline.

* *P <* 0.05;

** *P <* 0.01;

*** *P <* 0.001.

Abbreviation: IFA, iron and folic acid.

1 Model also accounted for cumulative IFA consumption, squared to address nonlinearity of the association.

2 Red meats, such as beef, lamb, goat, or wild game and organ meats.

3 Other animal-source foods, including eggs, poultry, and fish

4 Dark green leafy vegetables, legumes, nuts, and seeds.

5 Cereal and beverages fortified with iron Milo, Ovaltine, Cerelac, Yumvita, or Nido.

6 Citrus fruits, including oranges, lemons, pineapple, mango, etc. This dietary variable was measured over the previous 24 hours.

## Abbreviations used:

BMIZ: BMI-for-age z-score
GIFTS: Girls Iron–Folic Acid Tablet Supplementation
GLMM: generalized linear mixed model
Hb: hemoglobin concentration
IFA: iron and folic acid
JHS: junior high school
RCS: restricted cubic spline
SHS: senior high school
