# Supplementary material for: A School-Based Weekly Iron and Folic Acid Supplementation Program Effectively Reduces Anemia in a Prospective Cohort of Ghanaian Adolescent Girls
Source: J Nutr. Author manuscript; Available in PMC 2022 Jun 1. (PMC8172428; doi:10.1093/jn/nxab024)
Supplement: Supplemental Table 1 [file NIHMS1687163-supplement-Supplemental_Table_1.docx]

| Supplemental Table 1 (Online Only): Adjusted Change in Anemia Prevalence Difference and Hemoglobin Concentration Mean Difference from Baseline to Follow-on among Adolescent Schoolgirls in the Northern and Volta Regions of Ghana by Participant Characteristics | | | |
| --- | --- | --- | --- |
|  | Change in Anemia (p.p.) ^2^ | | Change in  Hemoglobin concentration (g/dL) ^3^ |
| Variable | Adjusted Diff. (95% CI) ^4^ | Adjusted Diff. (95% CI) ^4^ | |
| *Demographics* |  |  | |
| Age, years | 0.7 (-1.1, 2.5) | -0.07 (-0.12, -0.02)** | |
| Wealth index (tertile) |  |  | |
| Middle vs. lowest | -6.5 (-12.6, -0.4)* | 0.00 (-0.17, 0.16) | |
| Highest vs. lowest | -13.9 (-20.2, -7.5)*** | 0.15 (-0.03, 0.32) | |
|  |  |  | |
| *School* |  |  | |
| Junior vs senior/vocational high school | -3.0 (-10.8, 4.8) | -0.20 (-0.41, 0.02) | |
| Rural vs urban | -5.9 (-13.3, 1.6) | 0.34 (0.13, 0.55)** | |
| Peri-urban vs urban | -12.2 (-19.6, -4.8)** | 0.43 (0.22, 0.64)*** | |
|  |  |  | |
| *Health* |  |  | |
| Positive vs negative malaria test ^1^ | -0.9 (-8.7, 6.8) | 0.16 (-0.05, 0.37) | |
| Overweight vs normal weight ^1^ | -0.1 (-6.9, 6.6) | -0.04 (-0.23, 0.15) | |
| Obesity vs normal weight ^1^ | -5.8 (-20.4, 8.7) | 0.09 (-0.32, 0.50) | |
| Practice geophagy vs non-practicing ^1^ | -6.1 (-12.5, 0.4) | 0.47 (0.29, 0.65)*** | |
|  |  |  | |
| *Diet (consumption in previous 24 hours)* |  |  | |
| Rich source of heme iron vs not consumed ^1^ | 3.6 (-3.2, 10.4) | -0.10 (-0.29, 0.09) | |
| Fair source of heme iron vs not consumed ^1^ | -2.9 (-10.2, 4.4) | 0.27 (0.07, 0.48)** | |
| Rich source of non-heme iron vs not consumed ^1^ | 0.2 (-6.5, 6.9) | -0.15 (-0.34, 0.03) | |
| Foods and beverages fortified with iron vs not consumed ^1^ | -4.2 (-10.2, 1.9) | 0.03 (-0.14, 0.21) | |
| Citrus fruits vs not consumed ^1^ | 5.2 (-1.0, 11.5) | -0.15 (-0.32, 0.03) | |
|  |  |  | |
| Cumulative number of IFA tablets consumed ^5^ |  |  | |
| 0 | -10.4 (-22.0, 1.1) | 0.20 (-0.13, 0.53) | |
| 1 – 10 | ref | ref | |
| 11 – 20 | -14.1 (-22.6, -5.5)** | 0.24 (-0.01, 0.48) | |
| 21 – 30 | -9.9 (-18.1, -1.7)* | 0.33 (0.10, 0.56)** | |
| >30 | -13.3 (-23.8, -2.8)* | 0.46 (0.17, 0.76)** | |
|  |  |  | |
| Note: Estimates are weighted to be representative of all eligible girls in the school. Estimates, 95% confidence intervals, and p-values are calculated from generalized mixed models using maximum likelihood estimation and account for clustering in school and intra-individual covariance.  *p<0.05; **p<0.01; ***p<0.001;  ^1^ Repeated measures used.  ^2^ Percentage point (p.p.) difference in the change in the population prevalence of anemia from baseline to follow-on (8 months).  ^3^ Difference (g/dL) in the change in the population average hemoglobin concentration from baseline to follow-on (8 months).  ^4^ Adjusted for other variables in the table including demographics, school, health, diet, and IFA tablets consumed.  ^5^ Anemia model p-value for trend: 0.024; Hb model p-value for trend: 0.029; Increasing categories of consumption may not correspond directly to decreases in anemia due to the curvilinear relationship; The reference category was 1-10 tablets because of the robust sample size compared to the zero-dose category. | | | |
